# Drosophila Models of Huntington's Disease Exhibit Sleep Abnormalities

**DOI:** 10.1371/currents.RRN1185

**Published:** 2010-09-29

**Authors:** Erin Gonzales, Jerry Yin

**Affiliations:** ^*^University of Wisconsin-Madison and ^†^UW-Madison

## Abstract

The complex pathology of neurodegenerative diseases presents a challenge to researchers who model the disease, and clinicians who treat patients. The identification of early, perhaps even prodromal, biomarkers is important for developing strategies to ameliorate disease progression. Sleep disturbances are a clinical feature of Huntington’s disease (HD) as well as a part of normal aging. Whether sleep dysfunctions in HD patients are epiphenomenal or central to the neurodegenerative disease process is unclear. We show that sleep fragmentation is shared among Drosophila transgenic models that express mutant forms of huntingtin (mHtt), and flies with RNAi-mediated knockdown of the endogenous gene (dhtt). Our data suggest that sleep disturbances in HD may represent loss of function in the endogenous dhtt gene and that sleep perturbations in Drosophila HD models present an opportunity for screening therapeutic interventions.

## **Introduction:**

Huntington’s disease (HD) is an autosomal dominant disorder caused by a polyglutamine repeat expansion in the N-terminus of the huntingtin protein (Htt) that results in myriad molecular and pathophysiological features, including mitochondrial dysfunction, intracellular trafficking defects, abnormalities of protein folding and/or processing, and the diagnostic motor and cognitive defects [1] [2].  Gross defects like huntingtin aggregation and nuclear accumulation are thought to represent intermediate to advanced stages of molecular pathology[3] . Identification of early markers of neurodegenerative disease that can be reliably diagnosed is therefore essential for the development of therapeutic strategies to delay disease progression. 

Among the behavioral changes in HD patients are specific sleep disturbances and alterations in sleep architecture [4]. Whether and how these disruptions relate to disease progression is an unresolved question, although the presence of sleep and circadian defects among multiple neurodegenerative diseases suggests that they reflect shared underlying cellular mechanisms[5] [6] [7].  The R6/2 mouse is a widely used HD model.  It has circadian defects, and pharmacological reversal of these partially ameliorates cognitive problems [8].  However mice are nocturnal and exhibit distinctive rest patterns from humans. *Drosophila* melanogaster is a crepuscular organism whose activity patterns resemble those of humans, with consolidated patterns of rest during the nighttime [9] [10] [11].  We show here that nighttime sleep is disrupted across multiple *Drosophila* HD models, and that knockdown of the endogenous fly homolog (dhtt) is sufficient to perturb nighttime sleep.  Our data suggest a role for dHtt in maintaining sleep.

### **Methods and Materials**

**Stocks and matings:** Homozygous virgin females carrying UAS-driven Htt transgenes pUAS-128Q FL [M36E2], pUAS-Htt128Q1-208 N-terminus [M64], pUAS-16Q FL [M28], or  UAS-dhtt RNAi 36204 were mated to neuronal driver G28 elav-Gal4/CyO males, generating (UAS-Htt transgene)/CyO or  (UAS-Htt transgene)/elav progeny used in sleep experiments. The balanced G28 elav-GAL4/CyO line was used to drive all UAS-transgenes used in this paper (as shown in Figure 1h) to ensure that each transgene was expressed in the same cells. The pUAS-128Q HttFL(s) [M36E2], pUAS-128Q Htt1-208 N-terminus [M64], and  pUAS-16Q Htt FL [M28] lines were generous gifts of Dr. J. Botas (Baylor College of Medicine) and are described in Romero et al., 2008. The homozygous viable UAS-dhtt RNAi line, 36204, was obtained from the Vienna *Drosophila* RNAi Center [12].  All matings and stocks were maintained at 22°C on cornmeal-molasses-agar food.

**Sleep experiments: **Virgin females were isolated within hours post-eclosion using CO2 anesthesia and placed in vials containing standard cornmeal-molasses-agar food in cohorts of 6-20 flies. Flies were socialized for 24-48 hours following selection before being placed in TriKinetics (Waltham, MA) monitors in incubators at 22°C. All monitoring was performed under 12:12 light:dark conditions at 22°C unless otherwise specified.  All experiments were performed with a minimum n=16.  Data in this paper show results that have been replicated at least once. All data shown are for a single, representative day of recording, the first full day following placement in recording apparatus, to allow 14-18 hours for recovery from anesthesia prior to recording.

**Analysis: **Activity data were collected by the *Drosophila* Activity Monitoring System (DAMS) developed by TriKinectics and analyzed using an Excel-based macro provided by Dr. P. Shaw (Washington University). [13] [14]. Data are expressed as averages + SEM. Statistical significance was determined by two tailed Student’s t-test; p-values of less than 0.05 are marked *; p-values < 0.01, **; and p-values < 0.005, ***.

### **Results**

Transgenic flies containing a UAS-driven full length human Huntingtin transgene with a 128Q polyglutamine expansion (128Q FL) were crossed to flies containing a balanced pan-neuronal GAL4 driver (elav-GAL4/CyO) to generate 128Q FL/elav and 128Q FL/CyO flies.  The 128Q FL/CyO flies, which lack the elav-GAL4 driver required for expression of the UAS-driven transgene [15], were used as controls. The same elav-GAL4/CyO driver line was used for expression of subsequent transgenes UAS128Q Htt1-208 N-terminus [M64], UAS-16Q FL, or UAS-dhtt RNAi 36204 to ensure that Htt transgenes were expressed in the same cells and to limit variability in expression levels.

Pan-neuronal expression of 128Q FL resulted in a selective effect on nighttime sleep (Figure 1) characterized both by overall nighttime sleep loss and fragmentation.  Sleep loss increased later in the dark period (Figure 1a) resulting in an overall decrease in nighttime sleep of approximately 15% when compared to CyO sibling controls (Figure 1b).  The number and duration of sleep bouts is used to assess the consolidation of sleep [13] [14].  The duration of average and maximum daytime sleep periods for both 128Q FL/elav flies and CyO controls (Figure 1c) were indistinguishable, although the number of day bouts was decreased in 128Q FL/elav flies, explaining their trend toward diminished daytime sleep.  Nighttime sleep, in contrast, was considerably more fragmented for flies expressing the 128Q FL transgene.  The duration of the average sleep bout, as well as the length of the maximum sleep periods, were diminished by 50% or more relative to sibling controls (Figure 1d) while the number of sleep bouts in 128Q FL/elav flies almost doubled (Figure 1d). Together with the shortened night sleep bouts, this showed that flies expressing a full-length polyQ expanded Htt transgene exhibit a nighttime sleep interruption and fragmentation resembling that seen in human HD patients [4] [5].

Normal Htt genes contain a polyglutamine coding sequence of fewer than 35 CAG repeats [16].  Expression of a full-length hHtt transgene containing a sub-pathogenic polyQ run, UAS-16Q FL, had no significant effect on nighttime sleep (Figure 1e, 1g) and showed subtle changes in daytime sleep bout number (Figure 1f) but not overall daytime rest or average bout length. The absence of significant differences between 16Q FL/CyO and 16Q FL/elav flies demonstrates that the alterations of nighttime sleep shown in Figure 1a-1d are due to expression of the polyQ-expanded 128Q FL transgene and are not attributable to the presence of elav-GAL4 alone or a "wild-type" Htt transgene. 

**Figure:**
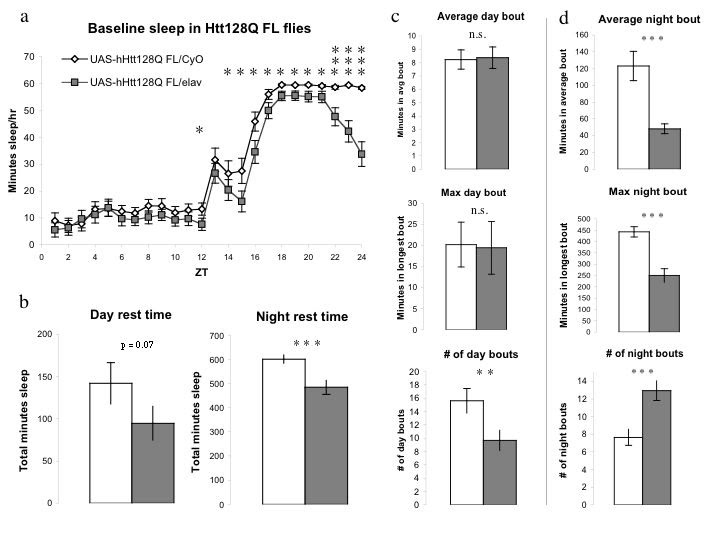

**Figure:**
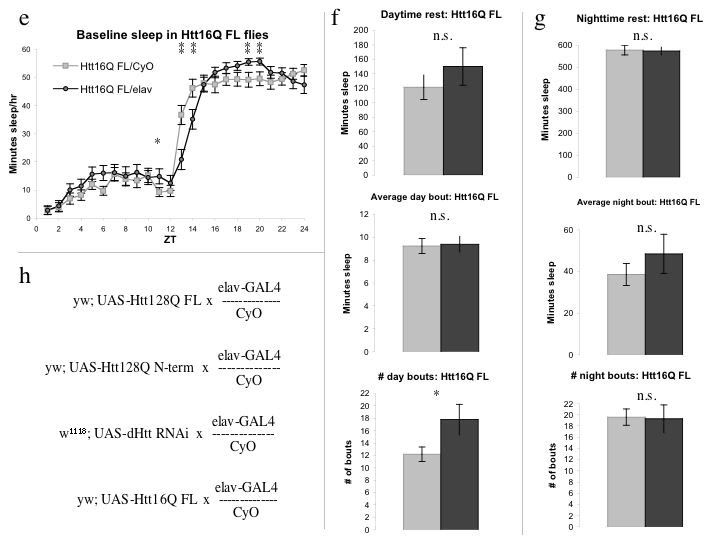

**Figure 1: UAS-hHtt128Q^FL^ flies display selective nighttime sleep deficits and fragmentation. 1a**, 128Q FL/elav flies exhibit sleep deficits throughout the night that become more pronounced over the dark period. **1b, c, d, **Day and night sleep are differentially affected by 128Q FL expression. Daytime sleep (**1b**), average day bout length, and maximum day rest bout (**1c**) are indistinguishable between groups, while number of day bouts in 128Q FL/elav flies is diminished. Total nighttime sleep (**1b**) is significantly reduced in 128Q FL/elav flies. **1d,** Duration of the average night bout and maximum night bout are dramatically decreased while number of night bouts in 128Q FL/elav flies is significantly increased in that in CyO siblings. **1e, f, g**, Flies expressing the Htt16Q FL transgene (16Q FL/elav) display very similar sleep patterns to their 16Q FL/CyO siblings, during both day (**1e, f**) and night (**1e, g**) periods. Nighttime rest is indistinguishable in amount and consolidation (**1g**) between 16Q FL/CyO and 16Q FL /elav flies, demonstrating that presence of elav-GAL4 alone has no effect on nighttime sleep. Day bout number (**Figure 1f**) shows a small but significant increase in 16Q FL/elav flies over CyO controls. **1h**, Mating scheme used for all experiments in the paper with female genotype on the left and male genotype on the right. Data are expressed as averages + SEM. Statistical significance was determined by two tailed Student’s t-test; p-values of less than 0.05 are marked *; p-values < 0.01, **; and p-values < 0.005, ***.

PolyQ-expanded N-terminal Htt models are the most widely used transgenic models of HD in rodents and flies. Previous groups [17] have used such an N-terminal HD model in flies as a cellular toxin to genetically ablate a very small subset of cells in the *Drosophila* brain (the sLNv cells) but the effects of expressing the transgene more widely were unknown. We analyzed a truncated version of the mutant human Htt protein containing a polyQ-expanded exon 1 (UAS-128Q N-terminal 1-208, or 128Q N-term) for sleep defects.  128Q N-term/elav flies displayed a notable increase in daytime sleep not seen in the 128Q FL flies, as well as a small but significant decrease in nighttime sleep (Figure 2a).

Nighttime sleep consolidation, however, varied strikingly between 128Q N-term/elav and 128Q N-term/CyO siblings with the primary difference being that 128Q N-term/CyO flies exhibit highly consolidated sleep (Figures 2h, 2i), while 128Q N-term/elav flies exhibit a much more fragmented sleep pattern of frequent short rest periods. The 128Q N-term/CyO flies exhibit normal crepuscular behavior with marked activity at “dawn” and “dusk” periods as measured by infrared beam breaks over the L:D cycle (Figure 2b) [10] [17], showing that the difference in consolidation of nighttime sleep is not due to hypoactivity in these animals. 128Q N-term /elav flies, in contrast, appear hypoactive, with little variation in activity over the day or night period (Figure 2b).  Given the abnormal nighttime sleep patterns and unusual activity of 128Q N-term/elav flies relative to their sibling controls, it was unexpected that their overall nighttime sleep was so similar. 

Daytime rest in 128Q N-term/elav flies is double that of CyO controls (Figure 2c).  The duration of average (Figure 2d) and maximum daytime (Figure 2e) sleep periods is not detectably different between groups, but the markedly increased number of daytime bouts (Figure 2f) alone accounts for the increase in daytime sleep. Nighttime sleep was (surprisingly) affected to a lesser degree than in the 128Q FL flies.  The N-term expanded flies had an overall decrease in night rest time (Figure 2g), diminished average night bout (Figure 2h) and maximum night bout (data not shown), as well as increased number of night bouts (Figure 2i). While fragmentation of nighttime sleep was still evident, expression of 128Q N-term pan-neuronally had a generalized effect on sleep and activity distinct from the night-specific effects seen in the full-length polyQ containing-flies. The flies also showed differential survival.  Although there was no detectable difference in survival between 128Q FL/elav and 128Q FL/CyO flies over three independent experiments (Figure 2j), there were obvious differences in mortality between 128Q N-term/elav and 128Q N-term/CyO siblings over the course of multiple experiments (Figure 2k). This is in accord with the findings of Sheeba et al. (2008) where the same transgene was used to eliminate the sLNvs. How the apparent toxicity of 128Q N-term expression may contribute to its generalized effects on sleep is unclear.

**Figure:**
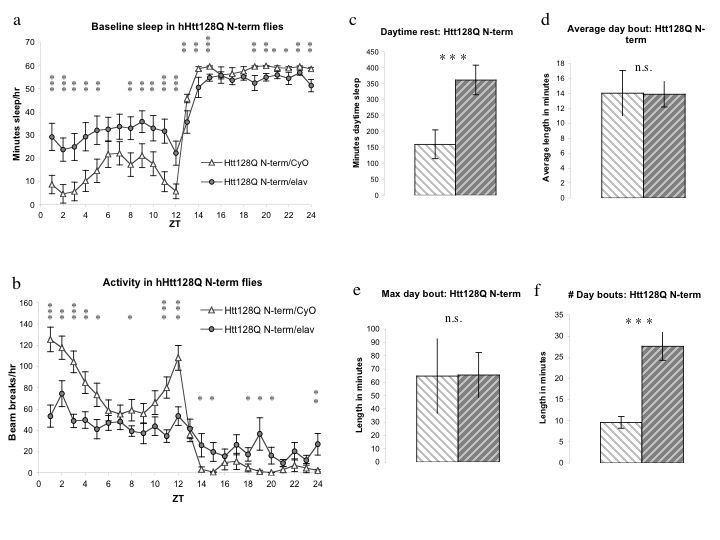

**Figure:**
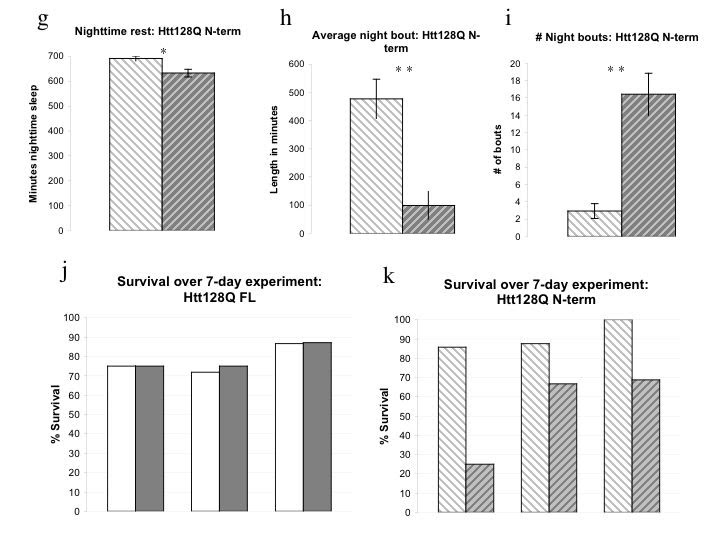

**Figure 2:** **UAS- hHtt 128Q N-term flies exhibit increased daytime sleep, decreased activity, nighttime sleep fragmentation, and decreased survival.  2a, **Flies expressing Htt128Q N-terminal^1-208^ fragment (128Q N-term/elav) display increased daytime sleep and decreased nighttime sleep within the first 5 days post-eclosion. **2b, **Activity is significantly reduced in 128Q N-term/elav flies across the L:D cycle, including at light:dark transitions. **2c,** Daytime sleep increased in 128Q N-term/elav approximately 2-fold over that of sibling controls lacking Gal4 driver. **2d, e, f, **Increased daytime sleep in128Q N-term/elav flies is due to increased number of bouts without significant change in duration. **2g,** Nighttime sleep in 128Q N-term/elav flies is decreased relative to CyO siblings. **2h, i,** Nighttime sleep in flies expressing 128Q N-term is marked by an increased number of shorter bouts. **2j, k, **Survival indices for three independent experiments with flies expressing 128Q FL (**2j**) or 128Q N-term (**2k**) showing decreased survival over seven days of recording in 128Q N-term/elav flies relative to CyO siblings that 128Q FL/elav flies do not exhibit.

Whether potential differences in expression between the UAS-128Q FL and UAS-128Q N-terminal transgenes contribute to the differences in sleep phenotypes is unknown. The two transgenes do display distinct patterns of aggregation and subcellular localization. The 128Q N-terminal transgene forms axonal aggregates not seen in the 128Q FL flies[18]. The 128Q FL transgene remains predominantly cytoplasmic while the 128Q N-terminal protein is concentrated in the nucleus [18]. This nuclear localization is common to N-terminal models [19] [20] and its role in pathogenesis remains controversial [20] [21].

Investigations using loss of endogenous Htt function in model organisms have yielded less of a consensus than overexpression studies using polyQ expanded N-terminal Htt transgenes. Conditional knockout of the essential mouse Htt homolog (Hdh) late in embryonic development or postnatally results in a progressive neurodegenerative state [22] [23].  Deletion of the nonessential *Drosophila* homolog dhtt resulted in decreased neural complexity, impaired motor function and diminished lifespan, and more rapid progression of Htt93Q N-terminal-induced pathological phenotypes [2]. Studies using RNAi knockdown of dhtt have confirmed the protective role of endogenous dhtt in the presence of a polyQ expanded Htt transgene, with an inverse correlation between the dhtt:mutant Htt ratio and the severity of the disease phenotype [24].  Examination of the endogenous functions of Htt represents an alternative approach to understanding HD pathogenesis [20] [25].

To determine whether dhtt loss of function might contribute to the sleep phenotypes seen in flies expressing 128Q FL and 128Q N-term transgenes, we used pan-neuronal RNAi knockdown of dhtt (UAS-dHtt RNAi VDRC# 36204). The degree of knockdown using dhtt RNAi has been estimated at 70-90% [26] but was not quantified for this work as it was not being driven universally. We found a subtle but significant effect on nighttime sleep in dHtt RNAi/elav flies (Figure 3a) resembling the 128Q FL sleep phenotype. Total sleep is diminished in dHtt RNAi flies (Figure 3b), an effect derived entirely from changes in nighttime sleep (Figure 3a, 3c-3g, and data not shown).  The fragmentation of nighttime sleep is evidenced by decreased average and maximum night bout (Figure 3d, 3e) and increased number of night bouts. This fragmentation of nighttime sleep is evident within the first five days of eclosion.  The data shown here is from the first full day of recording in a seven-day experiment. Given the generally normal neurodevelopment of dhtt null flies, the presence of this nighttime sleep deficit early in adulthood suggests a role for dhtt in maintenance of consolidated sleep.

**Figure:**
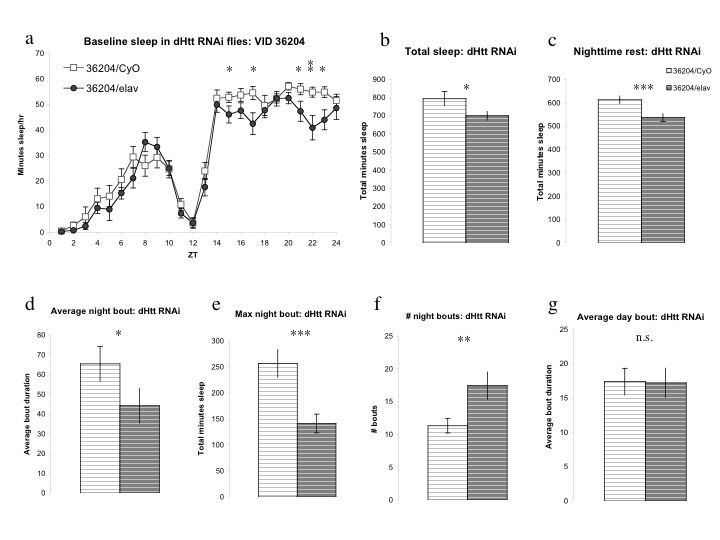

**Figure 3:** **RNAi knockdown of Drosophila Htt homolog dhtt results in selective nighttime sleep deficits and fragmentation. 3a**, UAS-dHtt RNAi (VDRC ID #36204)/elav flies exhibit mild sleep deficits that selectively affect nighttime sleep. **3b,** Total sleep in dHtt RNAi/elav flies is reduced relative to 36204/CyO siblings lacking Gal4 driver. **3c,** Nighttime rest is significantly reduced in flies expressing dHtt RNAi construct while daytime rest is indistinguishable between groups. **3d, e, f, **Average nighttime bout (**3d**) and maximum night bout duration (**3e**) are diminished while number of night bouts is increased, resulting in decreased consolidated nighttime sleep. **3g**, Daytime sleep is unaffected by expression of dHtt RNAi construct; average day bout (**3g**), daytime sleep (**3a**), maximum day bout duration and number of day bouts (data not shown) are indistinguishable between groups. 

Previous work characterizing the 128Q FL transgene showed reduced intracellular aggregation compared to the 128Q N-terminal transgene as well as an early neurotransmission defect [18]. Mortality and motor defects in flies expressing 128Q FL are delayed relative to those in 128Q N-term flies [27].  The expression of 128Q N-term is more toxic than that of a full-length protein when the polyQ expansion is held constant. The notable change in daytime sleep observed in 128Q N-term/elav flies may well be a product of such toxicity.  The defects in nighttime sleep are shared across all three models, and suggest a shared underlying mechanism such as a role for endogenous Htt in maintaining consolidated rest. Given that excitotoxicity is believed to be a significant contributing factor to HD pathogenesis [18] [28] [29], sleep disturbances in HD may be a systemic manifestation of an underlying pathology.

Both clinical data [30] [31] and work in transgenic HD models [2] [27] [26]  has shown that the interaction of mutant polyQ-expanded Htt with normal Htt is critical for determining the severity of disease phenotypes. The relevance of models that drive rapid pathophysiology or that recapitulate only the most dramatic defects of the complex pathology present in a post-mortem HD brain has recently been called into question [3]. Modeling early features or subtle phenotypes of HD is essential for developing strategies to delay onset or progression of neurodegenerative disease. The impairment of nighttime sleep shown here across three *Drosophila* models of HD mirrors the sleep disruptions seen in preclinical and early stage HD patients [4] [30]. Whether sleep loss represents a primary pathological process in HD remains an open question [4] [5] [6] [7] [31] but its presence across human patients and multiple models provides an opportunity for the assessment of therapeutic interventions.

**Acknowledgments:** E.D.G. would like to thank Eugenia Friedman for her comments on the manuscript.   **Funding Information:** Funding for this work was provided by a grant from CHDI to J.C.-P. Y. and UW-Madison AOF to E.D.G.     **Competing interests:** The authors have declared that no competing interests exist.     
